# Extracellular Glucose Increases the Coupling Capacity of the Yeast V H^+^-ATPase and the Resistance of Its H^+^ Transport Activity to Nitrate Inhibition

**DOI:** 10.1371/journal.pone.0049580

**Published:** 2012-11-26

**Authors:** Camila C. Ribeiro, Renan M. Monteiro, Flavia P. Freitas, Claudio Retamal, Layz R. S. Teixeira, Livia M. Palma, Flavia E. Silva, Arnoldo R. Façanha, Anna L. Okorokova-Façanha, Lev A. Okorokov

**Affiliations:** 1 Laboratório de Fisiologia e Bioquímica de Microrganismos, Centro de Biociência e Biotecnologia, Universidade Estadual do Norte Fluminense Darcy Ribeiro, Campos dos Goytacazes, Brasil; 2 Laboratório de Biologia Celular e Tecidual, Centro de Biociência e Biotecnologia, Universidade Estadual do Norte Fluminense Darcy Ribeiro, Campos dos Goytacazes, Brasil; Russian Academy of Sciences, Institute for Biological Instrumentation, Russian Federation

## Abstract

V H^+^-ATPase has an important role in a variety of key physiological processes. This enzyme is reversibly activated/partly inactivated by the addition/exhaustion of extracellular glucose. The current model of its regulation assumes the reversible disassembly/reassembly of ∼60–70% of the V1 and V0 membrane complexes, which are responsible for ATP hydrolysis and H^+^ conductance, respectively. The number of assembled complexes determines the pump activity because disassembled complexes are inactive. The model predicts the identical catalytic properties for the activated and semi-active enzymes molecules. To verify the model predictions we have isolated total membranes from yeast spheroplasts that were pre-incubated either with or without glucose. Nitrate treatment of membranes revealed the similar ATPase inhibition for two enzyme states, suggesting that they have identical structures that are essential for ATP hydrolysis. However, H^+^ transport was inhibited more than the ATPase activities, indicating a nitrate uncoupling action, which was significantly higher for the nonactivated enzyme. This finding suggests that the structure of the non-activated enzyme, which is essential for H^+^ transport, is less stable than that of the activated enzyme. Moreover, the glucose activation of the pump increases *i*) its coupling capacity; *ii*) its K_M_ for ATP hydrolysis and ATP affinity for H^+^ transport; *iii*) the Vmax for H^+^ transport in comparison with the Vmax for ATP hydrolysis and *iv*) the immune reactivity of catalytic subunit A and regulatory subunit B by 9.3 and 2.4 times, respectively. The protein content of subunits A and B was not changed by extracellular glucose. We propose that instead of the dissociation/reassociation of complexes V1 and V0, changes in the extracellular glucose concentration cause reversible and asymmetrical modulations in the immune reactivity of subunits A and B by their putative biochemical modifications. This response asymmetrically modulates H^+^-transport and ATP hydrolysis, exhibiting distinct properties for the activated versus non-activated enzymes.

## Introduction

The V H^+^-ATPases pump H^+^ from the cytosol across membranes for a variety of intracellular organelles, acidifying their lumen, and across the plasma membranes of specialised cells [1]–[10]. Among all H^+^ pumps, V H^+^-ATPase is the only type that has been shown to be involved in such different key physiological processes as H^+^ homeostasis, secondary transport of ions and nutrients, protein sorting, fusion/fission of membrane vesicles and the establishment of left/right asymmetry in vertebrates [1]–[11]. This enzyme has an important role in the proliferation of tumour cells [12], cell fusion [13], hyphal growth and the virulence of the human fungal pathogen *Candida albicans* [14]. The endosomal V H^+^-ATPase of the proximal tubule epithelial cells can regulate endocytic degradative pathways and modulate membrane trafficking by recruiting and interacting with the cytosolic GTPase Arf6 and the GDP/GTP exchange factor ARNO [15]. It has been suggested that the pump operates as a pH sensor, which may couple the intra-endosomal pH to the formation of endocytic transport vesicles [16].

**Figure 1:**
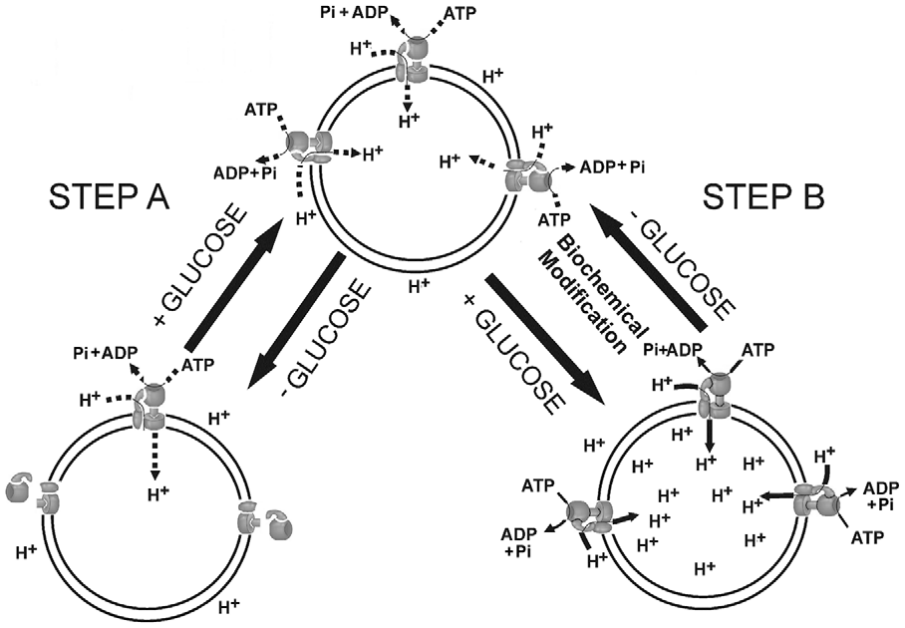
A model for the hypothetical regulation of V H^+^-ATPase activity by extracellular glucose. Step A: the dissociation and reassociation of the catalytic complex V_1_, according to [17], [18]; alternative step B: a putative biochemical modification and conformational change of V_1_ subunits (this report).

V H^+^-ATPase is reversibly activated/partly inactivated by the addition/exhaustion of extracellular glucose in yeast and animal cells, independent of its localisation to the vacuolar or plasma membranes [1]–[10], [17]–[19]. The modulation of its activity by extracellular glucose is similar to that found for the P H^+^-ATPase of the yeast *Saccharomyces cerevisiae* [2], [7], [9], [10], [17]–[22]. The modulating activities of both H^+^ pumps do not require new protein synthesis and occurs post-translationally in approximately 5 minutes [2], [7], [9], [10], [17]–[22]. However, the regulation of P H^+^-ATPase activity, an integral plasma membrane protein, is based on biochemical modifications, a change in conformation, ATP affinity modulation and the sensitivity to its inhibitor vanadate [20]–[25]. The regulation of V H^+^-ATPase activity is considered to be achieved via the complete physical dissociation/reassociation of ∼60–70% of the large catalytic V_1_ complexes from/with the membrane V_0_ complexes (Fig. 1, step A), which are required for proton transport [2], [10], [17]–[19], [20], [22]–[26].

This mechanistic model of the regulation of V H^+^-ATPase by extracellular glucose assumes that the number of V_1_ catalytic complexes bound to the membrane V_0_ complexes is the only determinant of enzyme activity [17], [18]. According to the model, in the presence of extracellular glucose, the V_1_ complexes would be bound to membrane and V_0_ complexes. However, when glucose is consumed, a significant fraction of the activated enzyme molecules (∼60–70%) becomes subject to dissociation of the V_1_ complexes from V_0_ and from the membranes. This dissociation causes a loss of activity of the V_1_ and V_0_ complexes, which results in residual enzyme activity of 30–40% (Fig. 1, step A). This model differs from the classical enzyme regulation model that is based on biochemical modifications and/or conformational changes in the enzymes. The model of the dissociation/reassociation of the V_1_ and V_0_ complexes assumes that the molecules of the activated and non-activated (semi-active pump) enzymes have the same properties, *i.e*. identical sensitivity to inhibitors, identical affinity to ATP and identical coupling of H^+^ transport and ATP hydrolysis (Fig. 1, step A). To test these predictions, we conducted several types of experiments. First, we compared the effect of different nitrate concentrations on ATP hydrolysis and H^+^ transport catalysed by the two enzyme states. As a chaotropic anion, nitrate destabilises enzyme structure, causing the dissociation of some subunits of the V_1_ complexes (catalytic subunit A and regulatory subunit B). The dissociation of these subunits inhibits both processes catalysed by the enzyme [26]–[28]. Because these nitrate effects were shown for the non-activated state of the V H^+^-ATPase and have not been analysed for the activated enzyme, it was important to know whether the two pump states would show identical properties with regard to the nitrate action as predicted by the current mechanistic model of the pump regulation by extracellular glucose. Second, the immune reactivity and protein content of subunits A and B were detected before and after treatment of the total membranes with 100 mM nitrate. Finally, the ATP affinity and Vmax of both enzyme states and the coupling between ATP hydrolysis and H^+^ transport were compared.

## Materials and Methods

### Yeast strain

Wild-type yeast *Saccharomyces cerevisiae* strain X2180 (*MATa SUC2 mal mel gal2 CUP1*) was a generous gift from Dr. L. Lehle (Regensburg University, Germany). The yeast cells were grown at 30°C in YPD medium containing 1% yeast extract, 2% bactopeptone and 2% glucose.

### Membrane isolation and fractionation

The yeast total membranes (TM) were isolated and fractionated according to [29]–[32]. Briefly, the middle logarithmic phase cells were transformed to the spheroplasts by incubation at 37°C in buffer containing 1.2 M sorbitol, 10 mM Tris–HCl, pH 7.4, 30 mM β-mercaptoethanol and 1 mg lyticase (Sigma)/1 g of wet cells. After 50 min of incubation, the mixture of spheroplasts, the old cells and cell walls were rapidly cooled and received EDTA, benzamidine and PMSF (phenylmethylsulfonyl fluoride) at 1.2 M sorbitol and 10 mM Tris–HCl, pH 7.4 to give a final concentration of 1 mM for each protease inhibitor. The resulting mixture was loaded on top of a 1.4 M solution of sorbitol in 10 mM Tris–HCl, pH 7.4 and centrifuged for 5 min at 3000×*g*. The pellets were softly re-suspended in a solution of 1.2 M sorbitol, 10 mM KH_2_PO_4_, 3 mM MgSO_4_, 10 mM Tris–HCl at pH 7.2 with or without 100 mM glucose. After a 10 min incubation at 37°C followed by a rapid change to 0–4°C, the mixture was centrifuged again, and the pellets were re-suspended in cold lysis buffer (12.5% sucrose, 20 mM MOPS-Na at pH 7.4, 1 mM DTT, 1 mM benzamidine, 1 mM PMSF and a cocktail of polypeptide protease inhibitors) with or without 100 mM glucose. The spheroplasts were then homogenised in a Potter glass homogeniser, and the TM were precipitated for 45 min at 120,000×*g*. The TM were re-suspended in lysis buffer without glucose, received the cocktail of the polypeptide protease inhibitors and were frozen before being used.

### Enzyme activities

To measure the H^+^ transport activity, membrane vesicles (3–45 μg of protein) were added to the incubation medium containing 50 mM KCl, 2.5 mM MgSO_4_, 12.5% sucrose, 20 mM Tris-Cl at pH 7.4 and 1 μM ACMA (9-amino-6-cloro-2-methoxyacridine). Different concentrations of nitrate were used to detect its inhibitory effect. H^+^ transport was initiated by the addition of 1 mM ATP-Na and was monitored by measuring the fluorescence quenching of ACMA [29], [30], [33] using a Shimadzu RF-530 1PC fluorimeter. The membranes were pre-incubated with 200–300 μM sodium vanadate for 3 min to block the H^+^ transport activity of P H^+^-ATPase. All residual H^+^ transport was inhibited by 10–22 nM concanamycin A. Low protein content was used to prevent a steady state of H^+^ transport greater than 45% of the fluorescence quenching.

ATP hydrolysis was determined by measuring the amount of P*i* released according to [34] with some modifications. In these assays, the linearity of the P*i* release was verified at least during 10 min by incubation at 30°C with 1 mM ATP-Na, 2.5 mM MgSO_4_ and 1 µM FCCP (cyanide *p*-(trifluoromethoxy)phenyl-hydrazone). The incubation medium received 0–200 mM NaNO_3_ to determine the nitrate inhibition of the V H^+^-ATPase hydrolytic activity. To stop ATP hydrolysis, the assay tubes were placed on ice after 10 min. Cold water was added to the reaction medium to give a final volume of 1 ml. Two ml of solution C, prepared by mixing (100∶1) solution A (0.5% ammonium molybdate, 2% H_2_SO_4_ and 0.5% SDS without Pi) and solution B (10% ascorbic acid), were added to the reaction medium for a total volume of 3 ml. The assay mixture was incubated at 30°C, and the P*i* released was estimated by measuring the blue phosphomolybdenic complex after 10 min at 750 nm. The V H^+^-ATPase hydrolytic activity was calculated as the difference between the values measured in the absence of nitrate and in the presence of its different concentrations. The difference calculated between the values obtained without and with 200 mM nitrate was taken as 100% activity of V H^+^-ATPase. Similar results were obtained when ATP hydrolysis was determined in the presence and absence of 250 mM sorbitol.

### Immunoblotting

The immune reactivity of subunits A and B was detected under conditions of low protein content to prevent a saturation of the cross-reactivity. TM proteins (5–15 µg) were separated by 10% SDS-PAGE, transferred to nitrocellulose membranes and probed with monoclonal antibodies specific for either subunit A (anti-H^+^-ATPase 69 kDa subunit 8B1-F3, Molecular Probes) or subunit B (anti-H^+^-ATPase 60 kDa subunit 13D11, Molecular Probes/Invitrogen) of yeast V H^+^-ATPase. Cross-reacting proteins were detected with peroxidase-conjugated secondary antibody (GE Healthcare). A density of the bands was detected as previously described [29]. To detect immune reactivity of subunits A and B in yeast membranes stripped with 100 mM nitrate TM (1 mg protein) isolated from spheroplasts, pre-incubated with or without 100 mM glucose were incubated for 60 min on ice with 100 mM KNO_3_, 5 mM ATP, 10 mM MgCl_2_, 1 mM EDTA, 10% glycerol, 2 mM DTT and 10 mM Tris-HCl (pH 7.5). The stripped membranes were dialysed at 4°C against a solution of 10 mM Tris-HCl (pH 7.5), 1 mM EDTA, 10% glycerol, 2 mM DTT and 0.1% β-mercaptoetanol. The membranes stripped with nitrate were precipitated for 30 min at 100.000× *g* and 4°C, re-suspended in 10 mM Tris-HCI (pH 7.5) containing 8 M urea, 5% SDS and 1 mM EDTA. The amount of protein loaded onto the gels was established in preliminary experiments using dot blot in order to detect immune reactivity similar to that of non-stripped TM.

## Results and Discussion

**Figure 2:**
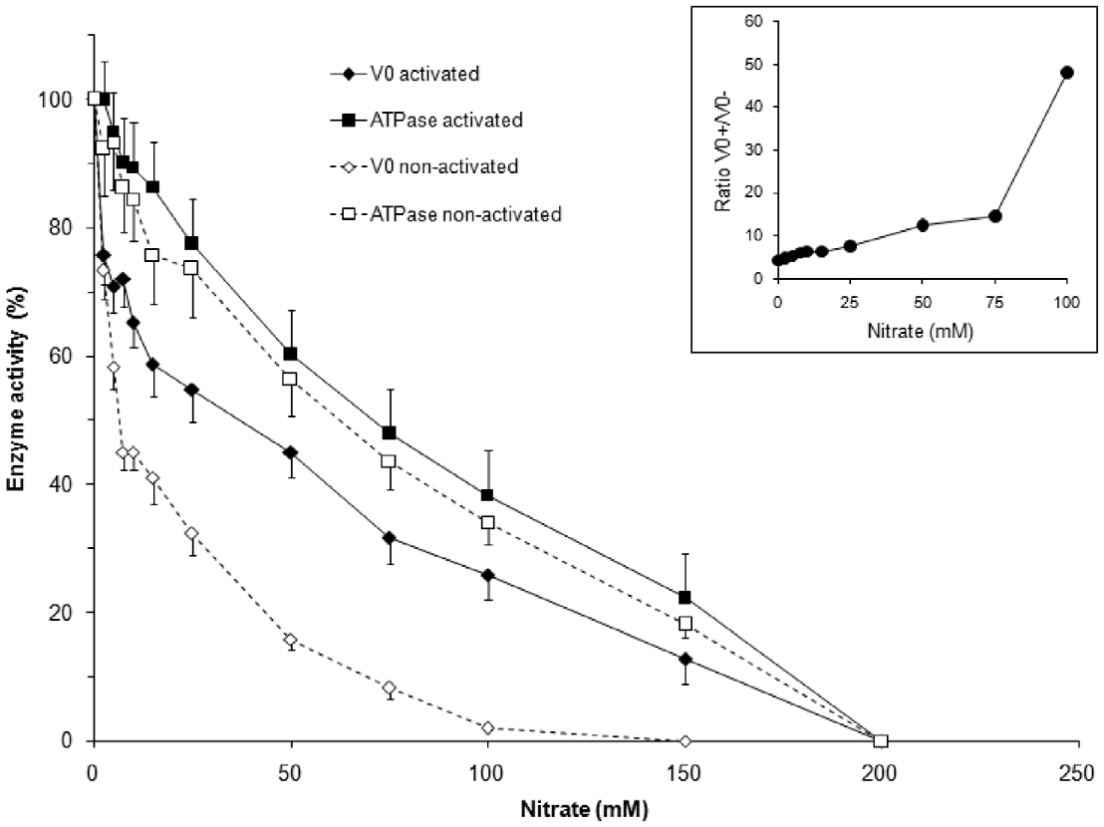
Nitrate inhibition of ATP hydrolysis and the initial velocity of H^+^ transport mediated by the activated and non-activated V H^+^-ATPase from the total membranes of *S. cerevisiae.* H^+^ transport was determined in the presence of 50 mM KCI to convert membrane potential to ΔpH. TM containing activated and non-activated V H^+^-ATPase were isolated from spheroplasts which were pre-incubated in a solution of 1.2 M sorbitol, 10 mM KH_2_PO_4_, 3 mM MgSO_4_, 10 mM Tris-HCl at pH 7.2 with or without 100 mM glucose, respectively (see Methods). The specific ATPase activities of the non-activated and activated enzymes were 0.76 and 0.46 µmol/mg ×10 min, respectively, whereas the initial velocities were 743±68 and 200±17 percentages of the fluorescent quenching/mg × min. *Insert*: The effect of nitrate on the ratio of the initial velocities of H^+^-transport catalysed by the activated enzyme state (V_0_^+^) and the non-activated state (V_0_^−^). The data are the mean values of at least seven independent experiments ± SE. In each experiment, the enzyme activities were determined in triplicate.

We first compared the nitrate sensitivity of the ATP hydrolytic activity of the activated and non-activated V H^+^-ATPase. Figure 2 shows that there was no statistically significant difference between these two states, with the enzyme activation rate changing from 1.2 to 3.0-fold. Because the enzyme shows ATPase activity only when the V_1_ complex is bound with the V_0_ complex and the membrane, our data suggest that both enzyme states display similar binding and/or interactions between the subunits of the V_1_ complex and its binding with the membrane. The enzyme structure, which is essential for its hydrolytic activity, appears to be very similar for the activated and non-activated enzyme states based on its destabilisation by chaotropic anions.

The data support the predictions of the current model for V H^+^-ATPase regulation by extracellular glucose regarding the catalytic identity of the activated enzyme molecules that did not lose their V_1_ and V_0_ complexes via dissociation and the ∼30–40% of activated enzyme molecules that remained after the hypothetical dissociation of the major part of the V_1_ complexes in the absence of extracellular glucose. These resting activated enzyme molecules represent the non-activated, semi-active state of the enzyme (Fig. 1, step A) [17], [18].

Based on the nearly identical sensitivity of the ATP hydrolytic activity of the two enzyme states to nitrate and the current view of pump regulation by extracellular glucose *in vivo*, H^+^ transport catalysed by the two pump states appears to be similarly inhibited by nitrate.

However, our data do not support this model prediction. Surprisingly, nitrate more efficiently inhibited the initial velocity of H^+^ transport mediated by the non-activated pump in comparison to that catalysed by the activated pump (Fig. 2). Compared to the activated pump, an approximately 6-fold lower concentration of nitrate was needed to decrease by 50% the initial velocity of H^+^ transport mediated by the non-activated pump (6–7 mM and 38 mM for the non-activated and activated pumps, respectively; Fig. 2). Similar results were observed for the steady-state transport of H^+^ (not shown). Thus, the structure responsible for H^+^ transport by the non-activated pump is not as stable as the activated state structure. This difference between the two enzyme states is greater when comparing the ratios of the initial velocities of H^+^ transport by activated and non-activated enzymes with increasing nitrate concentrations (Fig. 2, insert). The increasing ratio of the initial velocities illustrates the firmer structure of the residual activated enzyme molecules in comparison with the unstable structure of the non-activated pump molecules.

Nitrate inhibits the initial velocities of H^+^ transport by both enzyme states more effectively than their ATP hydrolytic activities (Fig. 2). This finding indicates that nitrate, even at low concentrations (but in the presence of 50 mM Cl^−^, a weaker chaotropic anion used here to transform membrane potential to ΔpH), uncouples ATP hydrolysis and H^+^ transport for the activated and non-activated enzymes. This uncoupling action by nitrate, which has been reported in a previous analysis of the non-activated enzyme of the chromaffin granule membranes from bovine adrenal glands [36], was less significant in the case of the activated enzyme (Fig. 2). This difference in uncoupling illustrates the differences between the two enzyme states, which contradicts the mechanistic model of V H^+^-ATPase regulation by extracellular glucose.

**Figure 3:**
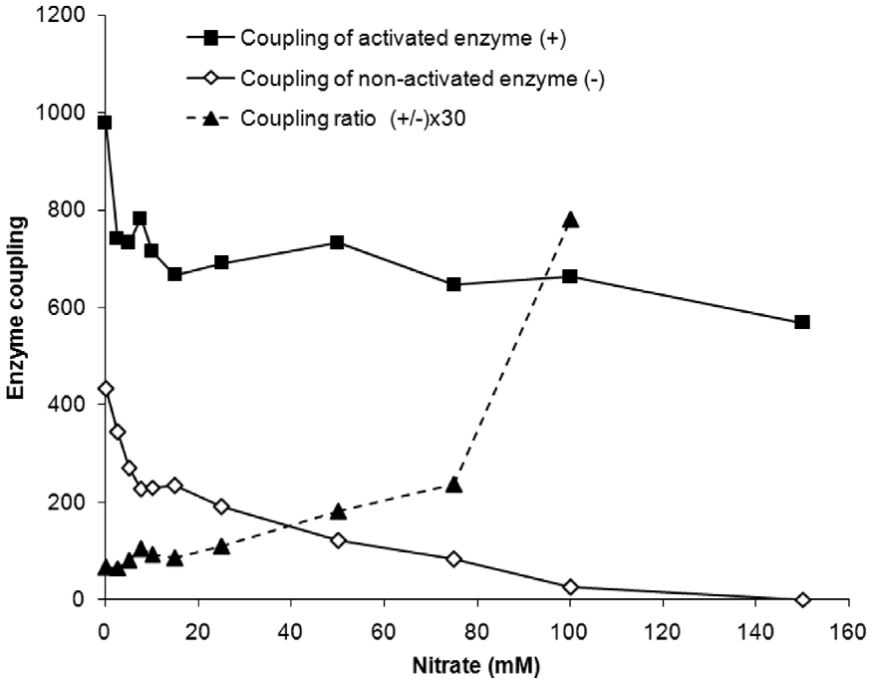
Nitrate inhibition of the coupling capacity of the activated and non-activated V H^+^-ATPase of the yeast TM revealed the higher coupling and higher stability of the activated pump. The coupling capacity (V_0_/ATPase) is given as a percentage of the fluorescent quenching/µmol ATP.

The coupling capacity of the pump was increased by extracellular glucose (Fig. 3). According to the model, the increase in the number of associated enzyme molecules must equal the growth in the activities of the two reactions catalysed by the pump (Fig. 1, step A), *i.e*. extracellular glucose cannot modify enzyme coupling. In contradiction to this prediction from the current hypothesis, our data on the coupling capacity of the two pump states revealed that it was at least as twice as high for the activated enzyme (Fig. 3). Furthermore, there was significant difference between the coupling capacities of the two pump states in their sensitivity to increasing nitrate concentrations (Fig. 3). Thus, the coupling of the activated enzyme was 26-fold higher than the coupling of the non-activated pump determined in the presence of 100 mM nitrate (Fig. 3). The coupling of the non-activated enzyme was diminished by 50% with 7.5 mM nitrate, whereas 100 mM nitrate reduced the coupling of the activated pump by only 32%. The H^+^ transport activity by the non-activated pumps was nearly abolished with 100 mM nitrate (the residual activity was 1.9%), whereas they preserved ∼34% of the ATPase activity. Therefore, the resting non-activated pumps were operating nearly in the “slip” (uncoupled) mode [35]–[37]. The change in the coupling ratio between the two reactions catalysed by the non-activated V H^+^-ATPase has been described for yeast and plant vacuoles [35]–[40]. This change depends strongly on the pH difference (ΔpH) across the vacuolar membrane, resulting in a ratio of ∼2 at high ΔpH (4 pH units), and increases to a ratio of 4 for low or near-zero ΔpH [38]. The variable coupling capacity is considered to be an important intrinsic property of the pump to respond to a variety of environmental conditions. Another factor that can significantly modulate the coupling capacity of the non-activated V H^+^-ATPase is the free Mg^2+^ concentration. Its change *in vitro* from very low values (1 mM Mg^2+^ATP is added to the incubation medium) to 1.0–1.5 mM (a typical value of free Mg^2+^ in cytosol [41]) can cause a significant stimulation of H^+^ transport by the non-activated pump [42]. Thus, an increase in free Mg^2+^ can stimulate the formation of membrane potential and ΔpH by ∼3 and 6-fold, respectively, whereas ATP hydrolysis is only increased by 10% [42].

The dependence of the coupling ratio on the ΔpH across the vacuolar membrane and its modulation by free Mg^2+^ was previously demonstrated *in vitro* under conditions in which the influence of factors such as the presence of microfilaments or a pool of free V_1_ complexes appear to be unlikely. These examples show that the H^+^ transport capacity of non-activated V H^+^-ATPase can be modulated to a greater extent than its ATPase activity, reflecting an intrinsic property of the pump.

It has not been previously reported that the coupling efficiency of V H^+^-ATPase *in vivo* is under extracellular glucose control or that the activated enzyme exhibits higher coupling rates than the non-activated pump. These findings were conclusively demonstrated in our experiments (Fig. 2 and 3). Furthermore, we are the first to report that H^+^ transport by the activated state of the V H^+^-ATPase displays higher resistance to the chaotropic agent nitrate, suggesting higher integrity and stability of the supramolecular pump complex in this state (Fig. 2 and 3).

**Figure 4:**
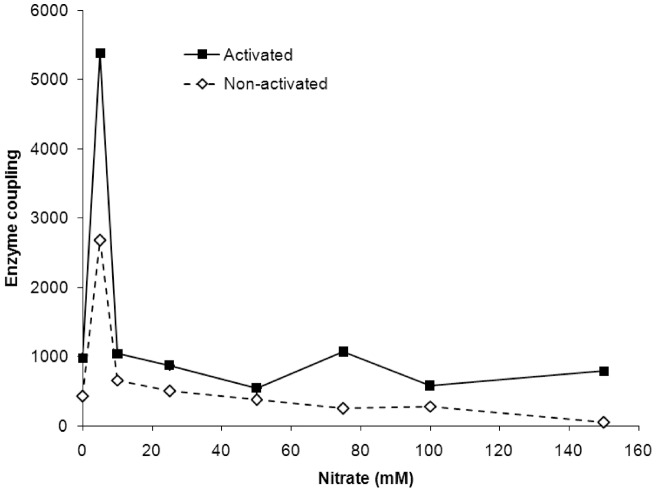
The coupling capacity of the sub-populations of the V H^+^-ATPase molecules inactivated by growing concentrations of nitrate. For each nitrate concentration, the coupling ratio was calculated by dividing the difference of the initial velocities of H^+^ transport (V_0_) for neighbouring concentrations by the similar difference in ATP hydrolysis. The enzymes exhibiting the highest coupling capacity of activated and non-activated states were inhibited by 5 mM nitrate in presence of 50 mM KCl. An example of the calculations: (V_0_ at 0 mM nitrate – V_0_ at 5 mM):(ATPase at 0 mM – ATPase at 5 mM).

A plausible interpretation of our data may be that the pump molecules endowed with better coupling are more resistant to the nitrate inhibition. However, this interpretation holds only when the activated and non-activated pumps are compared (Figs. 2 and 3). Surprisingly, the activated and non-activated pumps have small sub-populations of molecules that appear to be very sensitive to nitrate but are endowed with higher coupling capacity in comparison with the other pump molecules. Low nitrate concentrations significantly blocked the initial velocity of H^+^ transport by both enzyme states, whereas their ATP hydrolysis was inhibited only slightly (Fig. 2). For the activated pump, 2.5 mM nitrate decreased the initial velocity of H^+^ transport by 24% without any loss in its ATPase activity. Under the same conditions, nitrate decreased the ATPase activity and the initial velocity of H^+^-transport catalysed by the non-activated enzyme by 7.7% and 26.6%, respectively. At a concentration of 5 mM, nitrate inhibited 29% and 42% of the initial velocity of H^+^ transport catalysed by the activated and non-activated pumps, respectively. In contrast, the corresponding ATPase activities were only reduced by 5.3% and 6.7% for the activated and non-activated pumps, respectively. Interestingly, the coupling efficiencies of these small populations, which are found in both enzyme states and are inhibited by 5 mM nitrate, were 5.5 and 6.2-fold higher than the coupling efficiency for all of the pumps in the absence of nitrate (Fig. 4). Further increases in the nitrate concentration revealed that the resting enzyme molecules from the stripped membranes exhibited coupling efficiencies similar to that of the initial molecules from the total membranes (Fig. 4).

All of the enzyme molecules inhibited by the different nitrate concentrations showed similar coupling differences between the activated and the non-activated pump states, with values close to 2. The small sub-populations of the V H^+^-ATPase molecules (5–7%), which were selectively blocked by the 5 mM nitrate concentration and exhibited 5-6-fold higher coupling in comparison with the corresponding total pump populations, showed a similar trend. Future experiments should aim to identify the nature of this sub-population. Because the existence of this small sub-population does not depend on the presence of extracellular glucose, we speculate that it likely belongs to the same type of organelle(s). Given that the maximal coupling capacity was previously found for the pumps in membranes with very low ΔpHs [37], we further hypothesise that such a sub-population of pumps can either be localised to the *de novo*-formed membrane vesicles or represent pumps, with the greatest coupling efficiency from different organelles of unsynchronised cells growing at a maximal velocity.

The growing concentrations of nitrate increased the ratio of the initial velocities of H^+^ transport for the activated and the non-activated enzyme states (insert in Fig. 2). However, the ratio of the ATP hydrolytic activities was not changed significantly under the same conditions, suggesting an equal ratio between the number of catalytically competent V_1_ complexes and similar integrity for both enzyme states responsible for ATP hydrolysis (Fig. 2). Therefore, the increase in the ratio of the initial velocities of H^+^ transport with the increase in nitrate concentration can be explained by the different conformations/additional modifications of the activated and the non-activated enzymes. Moreover, this increase in the ratio reflects the higher sensitivity of the non-activated V H^+^-ATPase molecules to nitrate in comparison to the well-coupled molecules of the activated enzyme (Figs. 2, 3).

**Figure 5:**
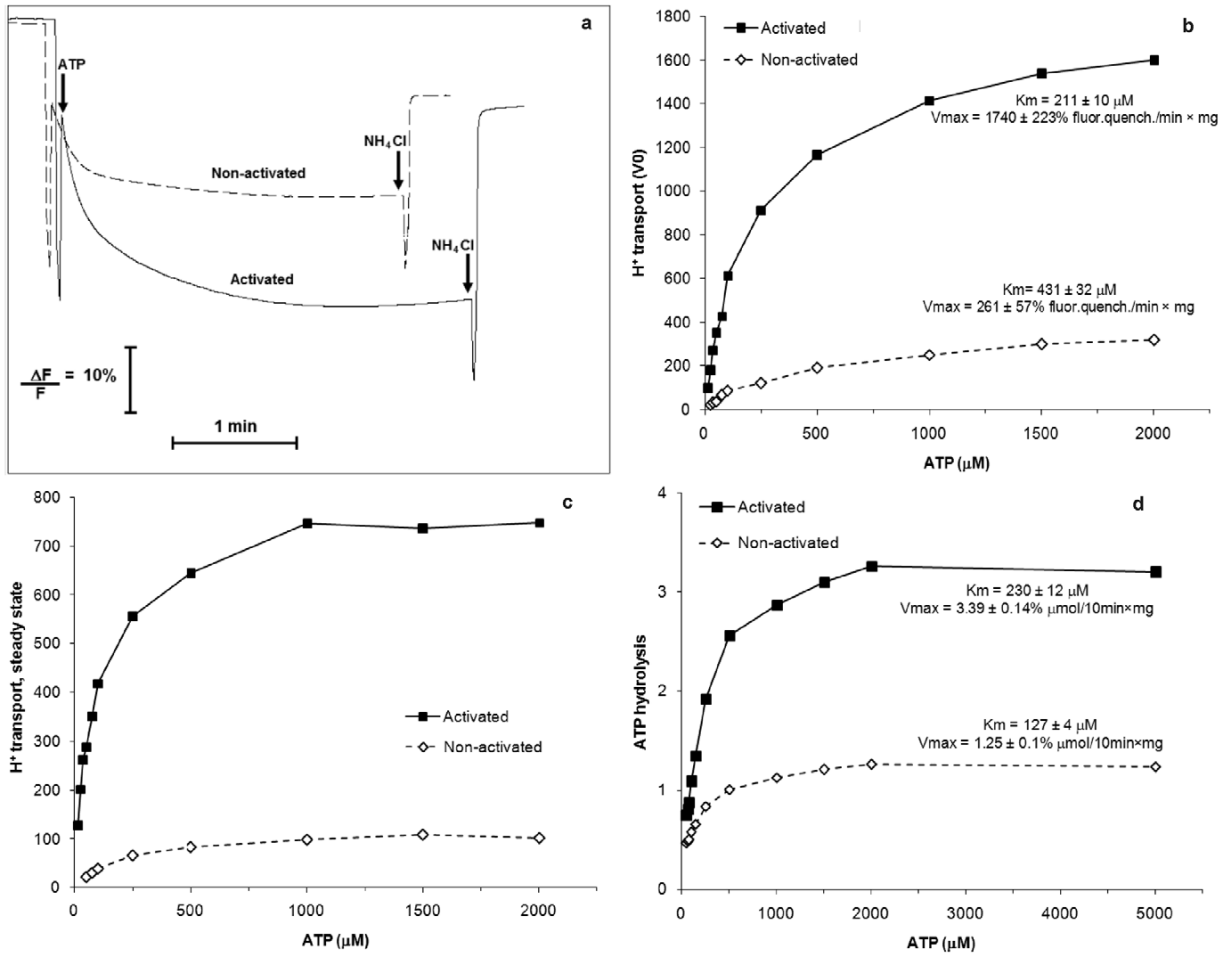
Activity of the V H^+^-ATPase in TM vesicles isolated from yeast spheroplasts pre-incubated in the absence or presence of 100 mM glucose. The H^+^ transport into the TM vesicles was determined using fluorescence quenching of ACMA in the presence of 1.0 mM ATP (a) or in the presence of 0.015–2.0 mM ATP (activated enzyme) and 0.025–2.0 mM ATP (non-activated enzyme) (b and c, respectively). The V_0_ of ATP hydrolysis (d) was determined by measuring the Pi release after 10 min of incubation. The V_0_ (b) and steady state (c) of the H^+^ transport are presented as a percentage of fluorescence quenching/min × mg of protein and as a percentage of fluorescence quenching × mg^−1^ of protein, respectively; the ATP hydrolysis is in µmol of Pi/mg protein ×10 min (for other details see “Methods”).

Therefore, we propose that the activated pump molecules exhibit a more solid structure. Our data on the increasing coupling capacity of the V H^+^-ATPase by extracellular glucose point to the uneven, asymmetric modulation of the Vmax for ATP hydrolysis and H^+^ transport. We carried out this comparison because the mechanistic model predicts an identical modulation of Vmax for the ATP hydrolysis and H^+^ transport (Fig. 1, step A), an identical ATP affinity for the two reactions catalysed by both enzyme states and because those enzyme properties have not been previously compared. Extracellular glucose increased the Vmax for ATP hydrolysis 2.7-fold as was expected from the mechanistic model; however, the Vmax for the H^+^ transport increased 6.7-fold, which is significantly higher than predicted by the model (Fig. 5 and Table 1). This asymmetric increase in Vmax ensures the modulation of the pump coupling capacity of at least 2.2- fold at 1 mM ATP in the absence of nitrate (Figs. 2 and 3) and 2.5-fold when the Vmax values are taken into consideration (Table 1). The effect of extracellular glucose on the ATP affinity for the two enzyme states was also selective. Although the K_m_ for the H^+^ transport increased with the decrease in Vmax in response to the glucose absence, its affinity for ATP hydrolyses increased (Fig. 5). This property of V H^+^-ATPase differs from that of P H^+^-ATPase, which decreased not only the Vmax but also the ATP affinity for ATP hydrolyses under a glucose deficiency [23]. This difference between the two H^+^ pumps is likely due to their physiological specificity under those conditions. Whereas P H^+^-ATPase has to energise PM to transport solutes during growth and cell division using protons supplied by glycolysis when glucose is available, it must decrease such activity when glucose is exhausted. The V H^+^-ATPase must continue its functions under glucose deprivation energising the secretory pathway membranes to ensure the secretion of the enzymes such as invertase, which are necessary to supply the cell with the carbon/energy source. Under these conditions V H^+^-ATPase has to increase its affinity for ATP, whose concentration is expected to be lower and to decrease Vmax because of the reduced production of its substrate, namely H^+^, when glycolysis is decreased.

**Figure 6:**
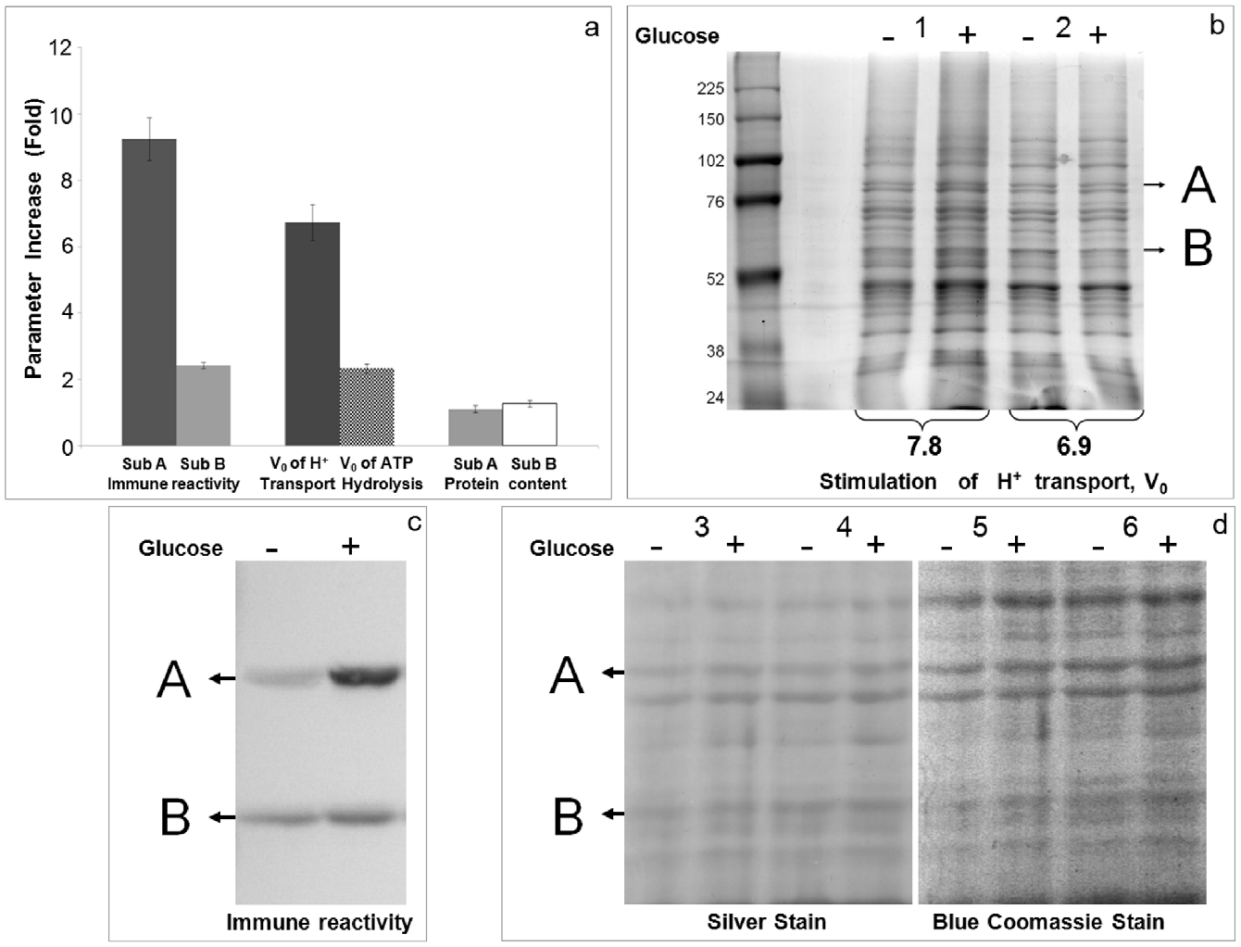
Comparison of properties of the V H^+^-ATPase activated and non-activated by extracellular glucose. (**a**) Extracellular glucose causes asymmetrical increase in the immune reactivity of subunits A and B as well as in the initial velocities of H^+^ transport and ATP hydrolysis. The protein content of these subunits was not changed in contradiction with the mechanistic model prediction (Fig. 1, step A). (**b**) Protein profiles of TM isolated from the spheroplasts pre-incubated with and without 100 mM extracellular glucose. The proteins were solubilised with 10% SDS, separated by SDS-PAGE (15 µg/each well; see Methods) and stained with Coomassie blue. The representative data from five independent isolations of TM are shown. The Arabic numbers indicate the stimulation degree of the initial velocities of H^+^ transport for each experiment. (**c**) Immunoblot of activated and non-activated enzyme; the amount of 30 µg and 15 µg of protein were loaded onto the wells corresponding to (−) and (+) glucose, respectively. (**d**) After SDS-PAGE, the protein bands were firstly visualised with Coomassie blue and, after gel destaining, were secondly detected by silver stain. Protein content loaded onto the wells 3−;3+;4− and 4+( = 5−;5+ and 6−;6+) was 7, 11, 13 and 15 µg, respectively. A merger of the Coomasie-stained gel and immunoblot was used to localise the bands corresponding to the subunits A and B in each experiment (not shown). One representative experiment of five independent membrane isolations is shown.

We sought to determine the change in the immune reactivity of catalytic subunit A and regulatory subunit B as a result of pump activation/partial inactivation depending on the extracellular glucose availability. The immune reactivity of those subunits in the TM not treated with nitrate was increased by 9.3 and 2.4 fold, respectively (Fig. 6, Table 1). The higher immune reactivity of catalytic subunit A of the activated pump correlated better with the increase in the initial velocity of H^+^ transport, whereas the modulation of the B subunit immune reactivity correlated better with the modulation of the ATP hydrolytic activity (Fig. 6 and Table 1). A noteworthy finding is the uneven increase in the immune reactivity of the catalytic and regulatory subunits of the enzyme by extracellular glucose (Fig. 6, Table 1). According to the current model, the increases in the immune reactivity of these subunits reflect the reassociation of the V_1_ holocomplexes with the V_0_ membrane complexes and, therefore, cannot be different from the 1∶1 ratio found in the V_1_ complex [17], [18]. The uneven modulation of the immune reactivity of subunits A and B found in our experiments contradicts the current model of enzyme regulation (Fig. 1, step A). However, the contradiction between our data on the different changes in the immune reactivity of subunits A and B and the prediction of the current model can be tentatively withdrawn assuming that after reassociation of the V_1_ complexes, the enzyme is subjected to the second step of its modification. This implies that biochemical modifications of the enzyme lead to the change in its conformation, coupling capacity, an asymmetric modulation of the immune reactivity of subunits A and B and other characteristics including differences in the enzyme kinetics and their sensitivity to nitrate inhibition (Figs. 1, step B; 2, 3 and 5). If this is correct, then one can expect an increase in the protein content of subunits A and B in the activated enzyme after the hypothetical reassociation of the V_1_ complexes (Fig. 1, step A). However, the analysis of the membranes isolated after the incubation of the spheroplasts with/without 100 mM glucose and the separation of their proteins in SDS-PAGE did not reveal any significant increase in the protein content in the bands corresponding to subunits A and B (Fig. 6 and Table 1). It is important to note that under the conditions of the stimulation of the initial velocity of H^+^ transport by 6.7-fold, the unchanged protein content was observed for subunit B, which had its immune reactivity increase by 2.5-fold, and for subunit A, which showed 9.3-fold increase. Simultaneously, the initial velocity of the ATP hydrolysis was stimulated by 2.7-fold, which is less than that of H^+^ transport (Fig. 5, 6 and Table 1).

**Table 1:** Changes in different characteristics of the yeast V H^+^- ATPase by extracellular glucose according to the current mechanistic model and experimental data of this study*.

	ATP hydrolysis		H^+^ transport		Coupling	Immune reactivity			Protein content	
	K_M_ *	Vmax	K_M_ *	Vmax	H^+^ transport/ATPase	Subunit A	Subunit B	Subunit A:B	Subunit A	Subunit B
Mechanistic model predictions	1	2.6**	1	2.6**	1	2.6	2.6	1	2.6	2.6
Experimental data	**1.8**	2.7	**0.5**	**6.7**	**2.5**	**9.3**	2.4	**3.9**	**1.1**	**1.2**

* All changes in characteristics for activated enzyme state are related to the respective characteristics for its non-activated state taken as one unit.

** Increase predicted by model. The ratio of the specific enzyme activities of vacuole membranes is used when non-activated enzyme has 1.5 units/mg (38.5% of the activated enzyme) [18], since Vmax for two enzyme states are not available in literature. That corresponds to the increase for V max of activated enzyme and the immune reactivity and protein content of subunits A and B by 2.6 folds, when the conformational changes and epitopes availability are not modified by glucose according to the current model. The contradictions between experimental data and the predictions of the mechanistic model are in bold.

Because the inhibition of V H^+^-ATPase activities by nitrate is accompanied by the partial removal of subunits A and B from the V_1_ complex [26]–[28], we further compared the immune reactivity of these subunits in membranes before and after their treatment with 100 mM nitrate. To this end, the immune reactivity of both subunits was determined in isolated and stripped membranes using the respective monoclonal antibodies. These membranes still exhibited 34% and 38% of the ATPase activity of the non-activated and activated enzymes, respectively (Fig. 2). The content of subunit A was evaluated by immunoblotting to be 16.3±1.5% and 15.4±1.4% for the non-activated and activated forms, respectively. The content of subunit B after the nitrate treatment of membranes was 17.1±1.6% and 18.6±1.7% for the non-activated and activated forms, respectively, which was very close to that of subunit A (not shown, data of three experiments). The data demonstrate that nitrate treatment of the activated and non-activated enzymes produces similar inhibition of ATP hydrolysis and equal removal of its subunits A and B for both pump states. The immunoblot data are therefore in agreement with our suggestion that the enzyme structure, which determines its ATP hydrolytic activity, is similar for both enzyme forms (see above and Fig. 2).

Interestingly, the 16–17% of subunits A and B in the non-activated pump stripped by nitrate treatment provided it with only 1.9% of its initial velocity of H^+^ transport (Fig. 2). In contrast, 15–19% of residual subunits A and B in the stripped activated enzyme provided it with 26% of that activity. The data strongly suggest that the presence of subunits A and B is not sufficient for the proper catalysis of H^+^ transport. To carry out H^+^ transport, the enzyme requires interactions among several subunits and probably other factors. Therefore, we assume that this enzyme state can be achieved by changes in the conformations of the V_1_ subunits A and B and their interactions with other subunits and with the V_0_ complex. This process can modulate the ATPase activity and improve the efficiency of the H^+^ transport activities and the coupling capacity of the V H^+^-ATPase.

It remains unclear why the relatively low content of subunits A and B (15–20%) in the stripped enzyme can provide 34–38% of the ATPase activity of the activated and the non-activated states (Fig. 2). One possible explanation assumes a heterogeneity of the V H^+^-ATPase molecules, which is due to the asynchronous nature of the analysed cells. Further experiments may clarify this apparent contradiction between the relatively low content of subunits A and B and the high ATPase activity in the yeast total membranes. This phenomenon has been reported for the non-activated enzyme in yeast vacuole membranes when their treatment with 100 mM nitrate resulted in residual ATPase activity of 29% and residual subunit A content of 18.5% (our evaluation of the subunit A residual content shown in Figs. 5 and 7 in [26]).

Interestingly, the ratio of the immune reactivity for the activated and non-activated enzymes in the stripped membranes increased for both subunits in comparison with non-treated membranes. In the case of subunit B, the ratio increased from 2.5 in TM to 3.8–4.5 in the stripped membranes. In a case of subunit A the ratio increased from 9.3 in TM to 15–17 in the stripped membranes (not shown). This observation, taken together with the data presented in Fig. 3, suggests that an enrichment of the stripped membranes with activated enzyme molecules exhibiting higher immune reactivity for subunits A and B may explain the increased coupling ratio of the enzyme molecules resistant to 100 mM nitrate in those membranes (Figs. 2 and 3).

We further speculate that the step-wise phosphorylation/dephosphorylation of subunits A and B are the molecular mechanism for the dynamic change in the ATPase and H^+^ transport activities and the enzyme coupling capacity. The highest pump activities and coupling can be achieved by the phosphorylation of all of the A and B subunits, whereas their step-wise dephosphorylation would result in a decrease in activity and coupling. Minimal residual activities and coupling might be achieved by complete (or nearly complete) pump dephosphorylation under glucose exhaustion, which simultaneously should cause a conformational change in the V_1_ complex and its weaker binding to the membrane complex V_0_. This weaker interaction between the complexes may facilitate their disassembly under some conditions, including those of immunoprecipitation [18]. In this case, different factors, such as 140 mM Na^+^(K^+^)Cl^−^, DMSO, C_12_E_9_ detergent and antibodies, would facilitate further release of the V_1_ complex from V_0_ and the membrane *in vitro*, artificially mimicking *in vivo* dissociation. Future experiments will test this suggestion and may reveal whether phosphorylation/dephosphorylation and/or other biochemical modifications of the enzyme are involved in its regulation by extracellular glucose.

During the 17 years since the important finding that extracellular glucose regulates the activity of the V H^+^-ATPase, the possibility that this regulation is based on the reversible dissociation/reassociation of the V_1_ and V_0_ complexes [17], [18] has been an important area of investigation in this field. These investigations have been, to a certain extent, aimed at understanding the molecular basis of V_1_ and V_0_ dissociation and reassociation. Some questions have not been addressed, discussed or resolved experimentally. First, how can the enzyme be rapidly re-activated by the diffusion of the large V_1_ complex through the cytosol? How can the possibility of V_1_ relocalisation from one organelle to another be explained given that it was considered as an advantage of the dissociation/reassociation model [18]. Such a possibility contradicts the recognised specificity of the V H^+^-ATPases for the distinct organelles established during their evolution. Lastly, what precise and effective mechanism determines why and how approximately 30–40% of all of the activated enzyme molecules can retain their activity and their V_1_V_0_-assembled structures while the remaining 60–70% experience dissociation of the V_1_ complex from the membrane complex V_0_, which leads to a loss in activity? Given that, according to the dissociation/reassociation model, only the change in the number of catalytically identical enzyme molecules determines the activity, resolving such a problem of selection between identical molecules appears difficult. One approach to address the last question is the suggestion that V H^+^-ATPase expressed in the Golgi/endosome [43] is not susceptible to V_1_ dissociation [44] and, therefore, the Golgi pump is not regulated by glucose *in vivo* by the same mechanism as the vacuole pump. However, this suggestion was based on results obtained with the Golgi enzyme isoform artificially relocalised to the vacuolar membrane (see discussion in [29]). The Golgi enzyme isoform exhibited a low initial velocity of the H^+^ transport (only 1.8%) when it was re-targeted to the vacuole membrane, lacking its own enzyme isoform [44]. The native V H^+^-ATPase activity and its coupling in the Golgi membrane is comparable or even higher in comparison with the native vacuole isoform [29], [30]. Moreover, we have recently found that the Golgi isoform of V H^+^-ATPase, localised to the Golgi membrane, is regulated by extracellular glucose (manuscript in preparation), which implies that the enzyme has to undergo V_1_ dissociation/reassociation, according to [17], [18].

Our study suggests that V H^+^-ATPase regulation by extracellular glucose cannot be adequately explained by the dissociation/reassociation of the V_1_ and V_0_ complexes. We suggest that the biochemical modification(s) of the enzyme subunits and the dynamic interactions between the subunits of the V_1_V_0_ complex and other enzymes/molecular complexes should be considered as the key factors affecting the selective modulation of the H^+^ transport and ATPase activities.

There are several possible factors involved in these modulation(s). A decrease in cytosolic pH as a result of glycolysis can be a signal for enzyme activation [45]. The subunits of the V_1_ complex can be phosphorylated or bound to microfilaments or RAVE complexes [2], [3], [8], [9], [46]–[50], [51]–[60]. It is also possible that an effect caused by a single modification of only one subunit may result in a domino-like effect within the hetero-subunit enzyme complex. The data indicating that different enzyme subunits are involved in its regulation also indicate the variety of specific mechanisms for fine enzyme regulation that are necessary because distinct organelle (iso)forms are differentially involved in key physiological processes.

In conclusion, our data indicate that the mechanism underlying the regulation of the V H^+^-ATPase by extracellular glucose *in vivo* is more complex than was previously envisaged by the mechanistic model, which assumes that a change in the number of pump molecules with identical catalytic properties can explain the different activities of the enzyme. Our results indicate different catalytic properties, including the different coupling capacity of the semi-active and the activated pump, the uneven modulation of the immune reactivity of subunits A and B and the different ATP affinities similar to the P H^+^-ATPase of the yeast plasma membrane [21]–[25], [60], [61]. The data also reveal a distinct inhibition of the two enzyme states by the chaotropic anion nitrate, which reflects the differences in their structures/conformations.
